# Travels of a Surgeon

**Published:** 1990-12

**Authors:** 


**TRAVELS OF A SURGEON**
**Arthur Eyre-Brook**
White Tree Books, Bristol £12.95

Arthur Eyre-Brook is an inveterate traveller. Since visiting the USA in 1937 as a Nuffield Scholar he has taken every opportunity to travel the world as a visiting Orthopaedic Surgeon, of latter years mainly in the third world. He has visited and worked, sometimes for long periods in Southern Africa, in the South West Pacific in Fiji and Tonga, in Brazil and Chile, in Kuwait and Iraw, in the Sudan, Malaysia, Burma, Bangladesh and Malawi. From all these countries he gives an account of the background of history and geography, of their antiquities, their social and political developments, and a wealth of information of all kinds on their people, their manners and customs. In view of the current crisis in the Gulf his account of a visit to Iraq is particularly interesting. He was there in 1969 when "there was a great deal of political tension, there had recently been four public executions in the main square, we were stopped frequently by the army when travelling. The publicised treason trial dominated the television and could be seen at all hours of the day. I was entertained many times at private houses and all eyes and ears were on the televised trial lest the names of anyone there present might be mentioned and threaten to draw them into the web of political intrigue. I have never had so much anxiety and even terror evinced around me."

The book is for the general reader and there is little mention of matters orthopoedic or even medical. It is as an account of travel in the wide world by an acute observer with a sharp eye and a memory for detail that the book will be most enjoyed.
M.G.W.

